# EGFR-targeted intraoperative fluorescence imaging detects high-grade glioma with panitumumab-IRDye800 in a phase 1 clinical trial

**DOI:** 10.7150/thno.60582

**Published:** 2021-05-21

**Authors:** Quan Zhou, Nynke S. van den Berg, Eben L. Rosenthal, Michael Iv, Michael Zhang, Johana C. M. Vega Leonel, Shannon Walters, Naoki Nishio, Monica Granucci, Roan Raymundo, Grace Yi, Hannes Vogel, Romain Cayrol, Yu-Jin Lee, Guolan Lu, Marisa Hom, Wenying Kang, Melanie Hayden Gephart, Larry Recht, Seema Nagpal, Reena Thomas, Chirag Patel, Gerald A. Grant, Gordon Li

**Affiliations:** 1 Department of Neurosurgery, Stanford University School of Medicine, Stanford, CA, USA.; 2 Otolaryngology-Head and Neck Surgery, Stanford University School of Medicine, Stanford, CA, USA.; 3 Stanford Cancer Center, Stanford University, Stanford, CA, USA.; 4 Department of Radiology, Stanford University School of Medicine, Stanford, CA, USA.; 5 Cancer Clinical Trials Office, Stanford University School of Medicine, Stanford, CA, USA.; 6 Department of Neuropathology, Stanford University School of Medicine, Stanford, CA, USA.; 7 Department of Neurology, Stanford University School of Medicine, Stanford, CA, USA.

**Keywords:** High-grade glioma, epidermal growth factor receptor, panitumumab-IRDye800, fluorescence imaging, phase 1

## Abstract

**Rationale**: First-line therapy for high-grade gliomas (HGGs) includes maximal safe surgical resection. The extent of resection predicts overall survival, but current neuroimaging approaches lack tumor specificity. The epidermal growth factor receptor (EGFR) is a highly expressed HGG biomarker. We evaluated the safety and feasibility of an anti-EGFR antibody, panitumuab-IRDye800, at subtherapeutic doses as an imaging agent for HGG.

**Methods**: Eleven patients with contrast-enhancing HGGs were systemically infused with panitumumab-IRDye800 at a low (50 mg) or high (100 mg) dose 1-5 days before surgery. Near-infrared fluorescence imaging was performed intraoperatively and *ex vivo*, to identify the optimal tumor-to-background ratio by comparing mean fluorescence intensities of tumor and histologically uninvolved tissue. Fluorescence was correlated with preoperative T1 contrast, tumor size, EGFR expression and other biomarkers.

**Results**: No adverse events were attributed to panitumumab-IRDye800. Tumor fragments as small as 5 mg could be detected *ex vivo* and detection threshold was dose dependent. In tissue sections, panitumumab-IRDye800 was highly sensitive (95%) and specific (96%) for pathology confirmed tumor containing tissue. Cellular delivery of panitumumab-IRDye800 was correlated to EGFR overexpression and compromised blood-brain barrier in HGG, while normal brain tissue showed minimal fluorescence. Intraoperative fluorescence improved optical contrast in tumor tissue within and beyond the T1 contrast-enhancing margin, with contrast-to-noise ratios of 9.5 ± 2.1 and 3.6 ± 1.1, respectively.

**Conclusions**: Panitumumab-IRDye800 provided excellent tumor contrast and was safe at both doses. Smaller fragments of tumor could be detected at the 100 mg dose and thus more suitable for intraoperative imaging.

## Background

High-grade gliomas (HGGs) are the most common primary malignant brain tumors in adults and the leading cause of cancer-related deaths in children. Despite intensive treatments with surgery, radiotherapy, and chemotherapy, the prognosis remains poor 1, 2. Residual tumor volume after surgery predicts recurrence and worse patient survival. However, complete resection is often not possible even for those tumors uninvolved in eloquent cortex due to the diffusive nature of HGG and thus the challenge of distinguishing the infiltrative tumor margin versus normal brain 3. To improve HGG visualization and safe extent of resection, intraoperative neuroimaging techniques such as neuronavigation, intraoperative magnetic resonance imaging (iMRI), ultrasound, OCT imaging, stimulated Raman imaging and fluorescence-guided surgery have been developed 4-9. Of these modalities, only intraoperative fluorescence imaging has been able to provide real-time high-resolution identification of tumor tissue *in situ* over the entire surgical field, resulting in intuitive output interpretation for the operating surgeon, while commercially available optical imaging systems routinely used in the clinic are integrated with accessary channels to support fluorescence-based surgery in the visible and near-infrared (NIR) range 10, 11. Use of 5-aminolevulinic acid (5-ALA) as an intraoperative fluorescent marker was approved by the Food and Drug Administration (FDA) in 2017 and has been successful at visualizing tumor intraoperatively 12. To date, none of the prior imaging approaches utilizes tumor selective biomarkers and thus they have limited tumor specificity and imaging contrast 13. As the World Health Organization (WHO) classification of central nervous system tumors continues to incorporate molecular parameters into the definition of tumor entities, molecular targeted fluorescence imaging has an increasing potential to impact surgical decision-making and therapeutic efficacy in these patients 14.

Epidermal growth factor receptor (EGFR) protein overexpression is seen in the majority of HGGs and is implicated in tumor cell migration and aggressiveness 15-17. In a mouse glioblastoma (GBM) model, panitumumab-IRDye800 provided highly specific optical contrast in both tumor core and margin in a patient-derived xenograft with highly overexpressed EGFR 18. Our recent preclinical study demonstrated that even modest EGFR protein expression can be detected with fluorescence imaging of panitumumab-IRDye800 in orthotopic GBM xenografts 19. Tumor target-to-background ratios reached 19.5 *in vivo* and 7.6 *ex vivo* when only 19% of tumor cells displayed weak to medium immunohistochemistry reactivity. This agent is further advantaged by its promising pharmacodynamics as panitumumab-IRDye800 bound to EGFR positive rat glioma cells with higher affinity than the fluorescent EGFR ligand, EGF800 20. Moreover, the fully humanized EGFR antibody, panitumumab, has an improved safety profile compared to its chimeric counterpart, cetuximab 21. However, the heterogeneity in EGFR protein expression can vary by orders of magnitude in human HGGs, resulting in a major barrier to personalized medicine 22, 23. This is the first-in-human report of imaging HGG with a fluorescent EGFR antibody, panitumumab-IRDye800, among other molecular imaging strategies 9, 24, 25.

We particularly sought to evaluate patients with pharmacologically accessible lesions. A leaky blood-brain barrier is a prerequisite for the effective intratumoral delivery of antibody-sized imaging probes. A previous proof-of-concept study using cetuximab-IRDye800 showed effective probe delivery in gliomas with contrast enhancement on preoperative T1-weighted MRI scan, but not in non-enhancing low-grade gliomas 24. T1-contrast enhancement indicates compromised blood-brain barrier by malignant tumor invasion into blood vessels, which is characteristic of most HGGs. Therefore, we hypothesized that fluorescence-guided imaging using subtherapeutic doses of panitumumab-IRDye800 can safely and effectively detect brain malignancies in patients with contrast-enhancing high-grade gliomas.

## Methods

### Participants

This prospective open-label phase 1 dose-ranging study investigated the feasibility of using panitumumab-IRDye800 (IND119474) as an optical imaging agent to detect HGGs during surgical resection. Adults with suspected high-grade gliomas (i.e. supratentorial T1 contrast-enhancing tumors on diagnostic MRI scans) undergoing standard-of-care surgery were eligible for the study. Patients were not preselected based on their EGFR status to be included in the study. Participants required a life expectancy of more than 12 weeks with a Karnofsky Performance Status of at least 70% or Eastern Cooperative Oncology Group/Zubrod level 1 and had not received an investigational drug within 30 days. Those with prolonged QT interval on electrocardiogram, significant cardiovascular or liver diseases, previous infusion reactions to monoclonal antibody therapies, or abnormal hemoglobin, platelet or white blood cell counts were excluded. Subjects who were pregnant, breastfeeding or receiving certain antiarrhythmic agents were also ineligible.

This study was approved by Stanford University Institutional Review Board (IRB43179) and the FDA (registered with ClinicalTrials.gov, NCT03510208), in compliance with the Health Insurance Portability and Accountability Act and the clinical trial registration statement from the International Committee of Medical Journal Editors. Written informed consent was obtained from all participants. Adverse events were categorized according to the National Cancer Institute Common Terminology Criteria (Version 4.03) and were collected up to 30 days after panitumumab-IRDye800 infusion. Each adverse event was reviewed by the operating surgeon and principal investigator to determine its attribution to panitumumab-IRDye800 26.

### Radiologic Evaluation

Preoperative MRI protocol for glioma diagnosis included T1-weighted and T2-weighted imaging for all patients while functional MRI images were collected from three patients with eloquent cortex involvement for intraoperative language and motor mapping. The evaluation included T1-weighted MRI sequences before and after intravenous gadolinium administration with 1 mm slice thickness. To measure MRI contrast of tumors, post gadolinium T1-weighted images were assessed using OsiriX 11.0 (Pixmeo Sarl, Bernex, Switzerland) by a radiologist blinded to the fluorescence images. Circular regions of interest (ROIs) with 3.0 mm diameter were manually reproduced at the interrogation sites in the intraoperative neuronavigation images corresponding to exposed tumor with NIR fluorescence. To ensure the accuracy of ROI mapping between the two modalities within the constraints of intraoperative workflow, extensive documentation of the neuronavigation probe locations on NIR fluorescence images during each interrogation and the corresponding neuronavigation coordinates were recorded simultaneously. The neuronavigation probe has a circular cross section measuring 3.0 mm in diameter, which determined the smallest possible ROI size for each interrogation site. For contrast-to-noise ratio (CNR) of the ROI on T1+C images, the following equation was used: CNR = (mean signal intensity of lesion - mean signal intensity of normal white matter) / standard deviation of lesion signal intensity 27, 28. Extent of tumor resection was determined from the first postoperative MRI scan obtained with 48 hours after surgery which was evaluated by a board certified neuroradiologist (randomly assigned by the health care facility and blinded to the study involvement of patients) and the neurosurgeon. Gross total resection was defined as no evidence of residual enhancing lesion on postoperative MRI; 90 - 99%, and 70 - 89% reduction in tumor volume were considered near-total resection and subtotal resection, respectively 29.

### Panitumumab-IRDye800 conjugation

Panitumumab (Vectibix; 147kDa, Amgen, Thousand Oaks, CA, USA) and IRDye800CW-N-hydroxysuccinimide ester (1kDa; LI-COR Biosciences, Lincoln, Nebraska, United States) were incubated for 2 h at 20 °C in the dark 30. The dye-to-protein ratio of panitumumab-IRDye800 was on average 2.0 (range: 1.0 - 3.0) determined by absorption spectrometry (A_780_ / A_280_). Quality control of the conjugate included purity (99.0%), concentration (5.0 mg/mL), excitation/emission peaks (774 nm / 789 nm), EGF receptor binding activity (78.5% relative to unconjugated panitumumab), pH (7.4) and sterility (no growth). The drug was produced following current Good Manufacturing Practices by the Frederick National Laboratory (Frederick, MD, USA), as part of the National Cancer Institute's NExT Program, and transported in sterile vials to Stanford University, Stanford, CA, USA, under temperature-controlled conditions, and vials were stored and dispensed by the Stanford University Medical Center Investigational Pharmacy.

### Near Infrared Fluorescence Imaging

**Figure 1:**
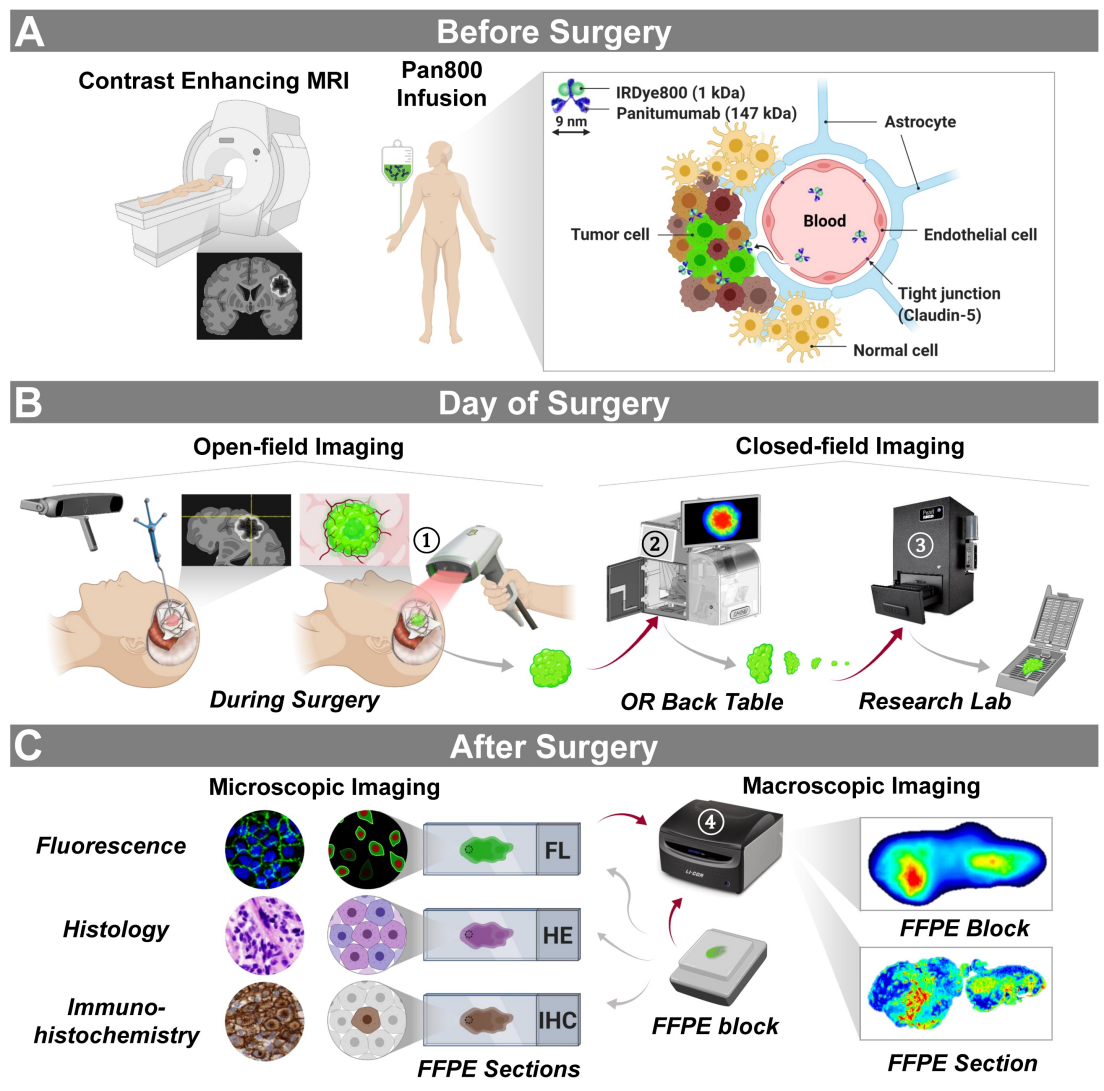
Trial design and imaging workflow. (**A**) Panitumumab-IRDye800CW was infused in patients with contrast enhancing high-grade gliomas prior to surgery. (**B**) Intraoperative near-infrared imaging of the surgical field with neuronavigation (open field) and resected tissue (closed field). OR: operating room. (**C**) Microscopic and macroscopic NIR fluorescence imaging of fixed tissue and corresponding histopathological stainings post surgery.* Red arrows*: tissue imaging path; *gray arrows*: tissue processing path. NIR fluorescence imaging instruments (vendors): ① SPY-PHI (Novadaq); ② IGP-ELVIS (Li-COR Biosciences); ③ Pearl Trilogy (Li-COR Biosciences); ④ Odyssey CLx (Li-COR Biosciences). Images reproduced with permission from Medtronic, Stryker, and LI-COR, Inc.

Patients with HGG received the study drug 1-5 days (according to the study IRB protocol) before surgery to accommodate the variability in presurgical logistics (**Figure 1A**). To identify the suitable dose for tumor detection, participants were alternatively assigned to receive either 50 mg or 100 mg of panitumumab-IRDye800. The dosages and timing of infusion were based on the previous study in HGG using cetuximab-IRDye800 24, as well as those in head-and-neck squamous cell carcinoma and pancreatic cancer using panitumumab-IRDye800 30-32. Compromised blood-brain barrier indicated by contrast enhancement on preoperative MRI scans allowed the fluorescent antibody to extravasate out of the blood vessels and bind to tumor cells expressing EGFR.

On the day of surgery, intraoperative open-field real-time near-infrared fluorescence images of the surgical field were detected using the SPY portable handheld imager with a light source customized for IRDye800 imaging (SPY-PHI, excitation/emission: 766 nm / > 800 nm; Novadaq, Burnaby, BC, Canada), first following tumor exposure, then during resection and again after completion of resection (**Figure 1B**). These open-field images are displayed in grayscale (fluorescence), heatmap (color segmented fluorescence) and overlay (fluorescence superimposed on white light) modes. The heatmap mode maps fluorescence intensities to a continuous range of colors in full saturation. Additional intraoperative images were acquired for one patient with a robotic operative microscope, KINEVO 900 (Carl Zeiss AG, Oberkochen, Germany), in white light and INFRARED 800 (excitation/emission: 700 - 780nm / 820 - 900nm; optimized for ICG visualization) modes 10, since ICG and IRDye800 have similar molecular constructs and compatible emission peak wavelengths (822 nm vs 789 nm) 33. The stereotaxic coordinates of individual interrogation sites in the brain were tracked with an intraoperative neuronavigation system (StealthStation^TM^ S8, Medtronic, Dublin, Ireland). Closed-field imaging devices, including the IGP-ELVIS (excitation/emission: 785 nm / > 820 nm, resolution: 85 µm; LI-COR Biosciences, Lincoln, NE, USA) and the Pearl Trilogy (excitation/emission: 785 nm / > 820 nm, resolution: 85 µm; LI-COR Biosciences), took NIR fluorescence images in an ambient light free environment 34. Tissue specimens, including en bloc primary tumors, resected tumor pieces as well as ultrasound aspirate and peritumoral tissue, were imaged *ex vivo* in the operating room immediately following removal, while tumor tissue further bisected were weighed and imaged in the research lab. Surgical specimens were subsequently formalin fixed and paraffin embedded (FFPE). Since this was a phase 1 safety study, the IRDye800 fluorescence information was not used to guide resection.

Fluorescence images of 4 µm thick sections from FFPE tissue blocks were scanned after surgery in a closed-field NIR imaging device, Odyssey CLx (excitation/emission: 785 / > 820 nm, resolution: 21 µm; Li-COR Biosciences 34), **Figure 1C**. To assess the distribution of panitumumab-IRDye800 in the tissue at cellular resolution, tissue sections were rehydrated, incubated in DyLight 488 labeled tomato lectin (10 µg/ml, Vector Laboratories Inc., Burlingame, CA, USA) for 30 min and counterstained with DAPI (4',6-diamidino-2-phenylindole, 300 nM; Invitrogen) before being examined under a custom-build NIR fluorescence microscope (DM6B, Leica Biosystems, Wetzlar, Germany; NIR excitation/emission: 774 nm / 789 nm 35). Image acquisition and processing was performed with LAS X software (Leica Biosystems). IHC staining and NIR imaging were performed on contiguous tissue sections, since some IHC reagents can quench the NIR signal. Based on the density of tumor cells in resected tumor and peritumoral brain tissue, regions of interest were assigned as “tumor” ( ≥ 95% tumor cells) versus “normal” (≤5% tumor cells) by two board-certified neuropathologists (H.V. and R.C.), who were blinded to fluorescence images, on corresponding H&E histology-stained tissue sections. To examine the expression of EGFR and other molecular biomarkers, automated immunohistochemistry (IHC) staining was performed on contiguous sections with Dako Autostainer (Agilent Technologies, Santa Clara, CA, USA) for EGFR (prediluted, RM-2111-RQ, Thermo Fisher Scientific, Waltham, MA, USA), Ki-67 (prediluted, GA62661-2, Agilent Technologies), Claudin-5 (1:500, 34-1600, Thermo Fisher Scientific) and ERG (1:1000, EPR3864, Abcam, Cambridge, United Kingdom). Secondary antibody: Envision FLEX+ rabbit (linker) (prediluted, SM805, Agilent Technologies) for EGFR; no secondary antibody was used for all other antibodies. Positive and negative controls were included in each staining batch. Immunoreactivity was visualized with diaminobenzidine and magenta chromogens (Dako EnVision, Glostrup, Denmark). Digital images of IHC-stained EGFR slides were obtained at 4 - 20 magnification with a whole slide scanner (NanoZoomer 2.0-HT slide scanner; Hamamatsu Photonics, Hamamatsu City, Japan).

### Image quantification

To measure intraoperative fluorescence imaging contrast, each *in vivo* SPY-PHI image was analyzed with ImageJ (version 1.53c). NIR fluorescence histograms (pixel intensity range: 0 - 255; pixel count range: 0 - 5000) of tumor and peritumoral normal brain areas were plotted from the intraoperative NIR grayscale images. Fluorescence intensities were quantified in circular ROIs (diameter: 50 pixels), corresponding to individual intraoperative interrogation sites with neuronavigation coordinates from the exposed tumor area in the surgical field and respective normal brain area in the same image. The following equation was used to calculate fluorescence CNR: CNR = (mean NIR intensity of lesion - mean NIR intensity of uninvolved brain tissue) / standard deviation of tumor NIR signal intensity. Fluorescence images of *ex vivo* tissue were quantified with the integrated instrument software ImageStudio (version 5.2, LI-COR Biosciences). Fluorescence heterogeneity was measured by standard deviation of fluorescence signal normalized by mean fluorescence intensity (MFI). MFI of fresh tissue was measured as total fluorescence signal divided by the total number of pixels within the region of interest. Target-to-background ratio (TBR) is defined as the MFI of primary tumor divided by the MFI of histologically negative tissue (i.e. peritumoral tissue samples removed for surgical exposure of the tumor and later confirmed to be histologically normal by a neuropathologist).

Annotation of tumor regions on slides was performed using Aperio's annotation software (ImageScope Viewing Software: Positive Pixel Count v9.1, Aperio ImageScope®; Leica Microsystems Inc.). The intensity of staining was graded as follows: negative, weak positive (Intensity Threshold weak [upper limit] = 220, [lower limit] = 175), medium ([upper] = 175, [lower] = 100), and strong ([upper] = 100, [lower] = 0) by default. The staining of EGFR was quantified by IHC positivity, which was calculated as the number of positive pixels stained at each positive intensity level divided by the total number of pixels (the number of positive and negative pixels) 36.

### Statistics

Data were expressed as mean ± SEM in text. TBRs against tissue size and weight were fitted (least squares) to logistic growth curves to identify projected thresholds of detection. Correlation between body weight adjusted dose and smallest detectable tissue weight was fitted to simple linear regression and goodness of fit was reported as R^2^. Receiver operating characteristics (ROC) curves were plotted for distinguishing histological tumor versus normal tissue using MFI as a diagnostic test. Sensitivity, specificity, area under the ROC curve, negative and positive predictive values were subsequently calculated using these definitions. Prism 8.4.1 (GraphPad Software, San Diego, CA, USA) was used for statistical analyses. One-way ANOVA analysis with multiple comparisons was conducted to compare means between groups in bubble plots (each symbol represents one patient) while median and interquartile range were indicated over each group. Paired t-test was performed to compare tumor CNR between preoperative MRI and intraoperative NIR in contrast-enhancing tumor areas (each symbol represents one individual interrogation site). Distribution of EGFR staining intensities between groups was compared by Chi-square test. Statistical tests for between group comparisons are specified in figure legends. P-value of 0.05 or less was considered statistically significant. (* *P* < 0.05; ** P < 0.01; *** P < 0.001; ns: not significant).

## Results

### Patient Population

Between May 2018 and September 2020, 19 patients were screened for enrollment of this phase 1 study. Of the 13 consented patients, two (15%) withdrew from the study before surgery, resulting in 11 patients completing the study. The workflow is outlined in **Figure 1**. Patients with suspected high-grade gliomas were recruited prospectively on the basis of supratentorial enhancing lesions on presurgical MRI consistent with a compromised blood-brain barrier (**Figure 1A**). They received either 50 mg (n = 5) or 100 mg (n = 6) of panitumumab-IRDye800 administered systemically 1 - 3 days before surgery, and the average time from infusion to surgery was 39 h (range:14 - 69 h). Fluorescence of resected tumor tissue as well as peritumoral tissue was measured in the operating room before the specimens were submitted for pathology triage.

The average age of enrolled patients were 56 years (range 32 - 72, **Table 1**). Craniotomy was performed to completely or partially remove tumors located in the frontal (45%), temporal (36%) and parietal (18%) lobes. Three (27%) patients underwent awake craniotomy for intraoperative language and motor mapping. Gross total resection was performed in two patients (18%), near-total resection in two (18%), and subtotal resection in seven (64%). Average maximum tumor diameter measured 4.4 cm. *De novo* primary tumors were found in seven (64%) patients without prior treatment history. Four (36%) patients had recurrent tumors with prior resection, chemotherapy and radiotherapy. On final pathology diagnosis and grading performed using the 2016 WHO criteria, glioblastoma (isocitrate dehydrogenase IDH1 wildtype, WHO Grade IV) was confirmed in ten (91%) patients. One (9%) patient was diagnosed with anaplastic oligodendroglioma (IDH1 mutation, WHO Grade III). None of the 41 adverse events (38 grade 1, two grade 2 and one grade 3) recorded were serious or attributed to panitumumab-IRDye800. No dose limiting toxicity events occurred in the two dose cohorts (**Table S1**).

### Intraoperative NIR fluorescence imaging of HGG

**Figure 2:**
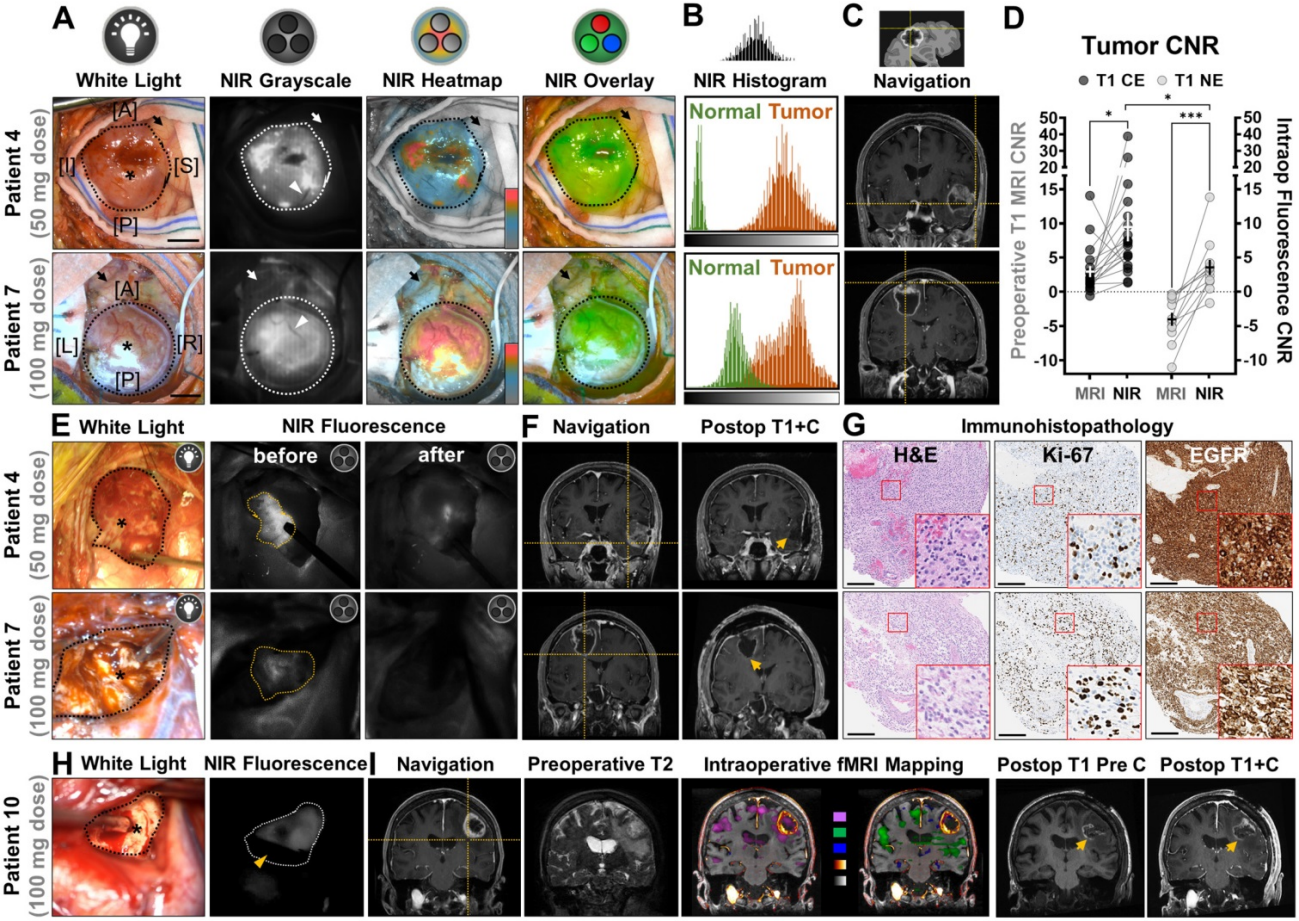
Intraoperative NIR fluorescence imaging of high-grade gliomas enhanced tumor contrast against normal brain in patients infused with either low (50 mg) or high (100 mg) dose of panitumumab-IRDye800. (**A**) Exposed tumors in the surgical field under white light and NIR fluorescence (in grayscale, heatmap and overlay modes, taken with hand-held imager). *Dotted outlines:* tumor margin; *arrows:* peritumoral brain tissue;* arrowheads:* blood vessels feeding into the tumors; *asterisks:* interrogation sites for neuronavigation. [A]: anterior; [P]: posterior; [I]: inferior; [S]: superior; [L]: left; [R]: right; Scale bars = 1 cm. (**B**) Histogram of NIR fluorescence (quantified from intraop NIR grayscale images) in tumor and peritumoral normal brain tissue. *X-axis*: pixel fluorescence intensity (range: 0 - 255); *Y-axis*: pixel count (range: 0 - 5000). (**C**) Neuronavigation coordinates (*crosshairs*) on presurgical contrast-enhanced MR images corresponding to interrogation sites in the surgical field. (**D**) Tumor contrast-to-noise ratio (CNR) improvement by intraoperative NIR imaging of tumor against peritumoral brain tissue, at interrogation sites within contrast enhancement (CE) and non-enhancing (NE) areas (*right y-axis*) compared to preoperative T1-weighted MRI scans of tumor against contralateral hemisphere white matter (*left y-axis*). Each symbol represents one intraoperative interrogation site. * *P* = 0.015 (CE MRI vs NIR), *** *P* = 0.0002 (NE MRI vs NIR) by paired t-tests and * *P* = 0.04 (CE vs NE NIR) by unpaired t-test. (**E**) Representative intraoperative white light photographs of resection cavities (*black outlines*) and corresponding open-field NIR fluorescence images (taken with hand-held imager) before and after removal of residual tumors (*yellow outlines*) to reach final wound bed. *Asterisks:* interrogation sites for neuronavigation. (**F**) Neuronavigation coordinates (*crosshair*) marking the location of the residual tumors on presurgical MR images and final wound bed (*arrows*) in post-operative contrast-enhanced MR image. (**G**) Pathological (H&E) and immunohistochemistry staining on residual tumor tissue against tumor proliferation marker, Ki-67, and EGFR. *Insets*: magnified bright-field microscopic views of cellular staining pattern. Scale bars = 200 µm. (**H**) Representative intraoperative white light photographs of wound bed (*black outline*) and corresponding open-field NIR fluorescence images (taken with surgical microscope) showing presence of residual tumor (*white outline*). *Asterisks*: interrogation sites for neuronavigation; *yellow arrowhead*: suction instrument for surface bleeding. (**I**) Neuronavigation coordinates (*crosshair*) marking the location of the residual tumors in the wound bed on presurgical MR images outside contrast-enhanced boundary in T1 MRI image. Edema and eloquent cortex involvement in peritumoral brain tissue on preoperative T2 image and fMRI mapping (P*ink*: visual responsive naming; *green*: tongue movement; *blue*: negative BOLD imaging signal; *glow heatmap*: T1+C;* grayscale*: pre-contrast T1), respectively. Postoperative T1 images of wound bed (*arrows*) before and after contrast injection.

The operating surgeon delineated the tumor borders in the surgical field under white light illumination and individual sites of exposed tumor were interrogated with a neuronavigation probe, as shown in an illustrative case from each dose cohort (**Figure 2A**). In an open-field imaging environment, strong NIR fluorescence (displayed in grayscale, heatmap and overlay modes) was detected within tumor areas with a hand-held imager, while minimal fluorescence was detected in the normal brain or vasculature feeding into the tumors. Despite slightly lower average overall tumor fluorescence in the 50 mg cohort (108.4 ± 30.0 vs 122.1 ± 22.1, *P* = 0.045), the contrast of NIR fluorescence in tumor against uninvolved normal brain tissue was more pronounced (4.7 ± 1.6 vs 2.3 ± 0.4, *P* = 0.038) than that of the 100 mg dose (**Figure 2B**). Intraoperative neuronavigation correlated the tumor fluorescence with preoperative T1-weighted MRI scans at individual interrogation sites (**Figure 2C**). Of the 19 intraoperative interrogation sites within the T1 contrast-enhancing (CE) margin of tumor obtained in all patients, the average fluorescence contrast-to-noise ratio (CNR) was a 4-fold increase (9.5 ± 2.1 vs 2.3 ± 0.8, *P* = 0.015) from the average preoperative MRI CNR of tumors (**Figure 2D**). Another 11 sites (91.7%, n = 12) in the non-enhancing (NE) area beyond the T1 CE margin on preoperative MRI, which were later confirmed as tumor on histology, had elevated tumor fluorescence CNR compared to T1 images (CNR = 3.6 ± 1.1 vs - 4.0 ± 0.9, *P* = 0.0002), but significantly lower than the CE sites (*P* = 0.04).

Although intraoperative NIR fluorescence imaging was not used for decision-making in this phase 1 study, NIR fluorescence imaging highlighted unsuspected, residual tumors in resection cavities with improved visual contrast**.** After residual tissue removal little fluorescence remained in the wound bed (**Figure 2E**). In two representative cases, intraoperative neuronavigation located residual tumor on the edge of contrast enhancement on preoperative MRI, but no contrast enhancement was found in the final wound bed on postoperative MRI, indicating complete resection of the contrast-enhancing portion of the tumor (**Figure 2F**). Immunohistopathology stainings of tissue biopsies confirmed presence of proliferative tumor cells, and strong positivity for EGFR (membranous and cytoplasmic) in the resected residual tumor tissue (**Figure 2G**).

To streamline the intraoperative fluorescence visualization within the surgical workflow, a neurosurgical microscope equipped with an appropriate filter (INFRARED 800) was adopted to detect NIR fluorescence simultaneously as ongoing resection (**Figure 2H**). Intraoperative neuronavigation located fluorescent residual tumor beyond the contrast enhancing component of the tumor where preoperative T2-weighted MRI scans and intraoperative fMRI mapping indicated peritumoral abnormality and involvement of eloquent cortex for language and motor functions at the interrogation site, respectively. Hyperintense T1-weighted signal present in the wound bed in post-operative contrast-enhancing T1 images was attributed to blood component rather than contrast-enhancing tumor, as such hyperintensity was present before the gadolinium-based contrast agent administration (**Figure 2I**).

### Threshold of detection

**Figure 3:**
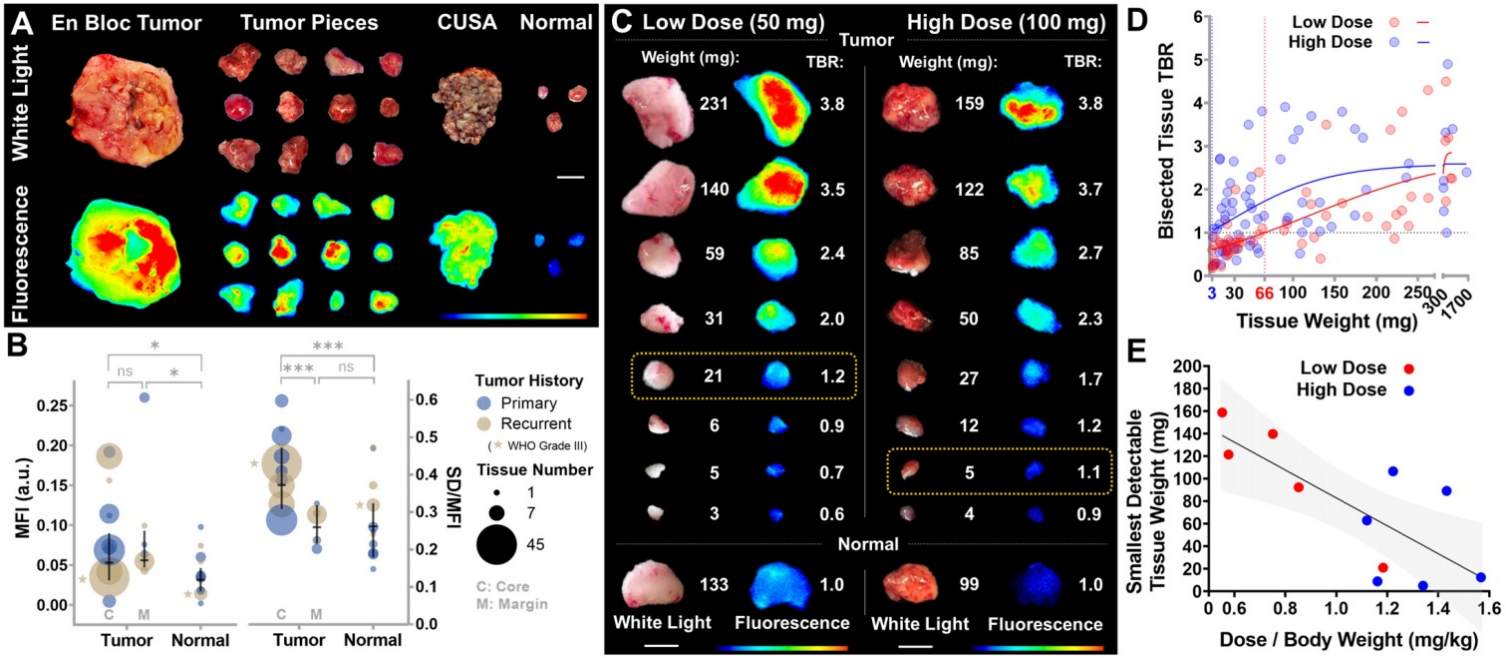
Detection thresholds of intraoperative NIR fluorescence imaging *ex vivo*. (**A**) Intraoperative white light photographs and corresponding closed-field NIR fluorescence images of resected tissue. CUSA: cavitron ultrasonic surgical aspirator. Scale bar = 1 cm. (**B**) Average mean fluorescence intensity (MFI) and fluorescence heterogeneity (standard deviation normalized to mean fluorescence intensity) in tumor and normal tissue pieces. Each circle represents one patient. MFI: *P* = 0.7407 (C vs M), * *P* = 0.0364 (C vs Normal) and * *P* = 0.045 (M vs Normal); SD/MFI: *** *P* = 0.0003 (C vs M) and *** *P* = 0.0001 (C vs Normal) by ANOVA with multiple comparisons. (**C**) Postoperative white light photographs and corresponding closed-field NIR fluorescence images of serial bisected fresh tumor tissue *ex vivo*. *Yellow boxes*: smallest detectable tumor tissue in each dose cohort. Scale bars = 1 cm. (**D**) Target-to-background ratios (TBR) of resected tissue fluorescence is plotted against tissue weight of each dose cohort (each symbol represents one tumor piece). *3 mg and 66 mg:* intersections of fitted curves with TBR = 1. (**E**) The smallest detectable tumor tissue weight correlated with body weight adjusted panitumumab-IRDye800 dose. *P* = 0.007 (each symbol represents one patient, simple linear regression goodness of fit: R^2^ = 0.57).

Immediately following removal, resected tissue specimens were imaged *ex vivo* in the operating room (**Figure 3A**). Fluorescence signal in tumor tissue taken from the contrast enhancing core was both higher (0.08 vs 0.04, n = 143 vs 23, *P* = 0.04) and significantly more heterogeneously distributed than normal brain tissue (0.39 vs 0.27, n = 143 vs 23, *P* = 0.0003,** Figure 3B**). While specimens taken in the tumor margin beyond the T1 contrast-enhancing area showed similarly increased fluorescence signal intensity as the core (0.08 vs 0.09, n = 143 vs 20, *P* = 0.74), their fluorescence heterogeneity was not significantly different from normal brain tissue (0.27 vs 0.27, n = 20 vs 23, *P* = 0.99). To investigate the size and dose dependence of tumor detection, specimens after pathology triage were further bisected and weighed post-operatively. As little as 21 mg (low dose) and 5 mg (high dose) of tumor tissue were fluorescently detectable (TBR > 1,** Figure 3C**). Among resected tumor pieces, 86% (n = 162) were detectable via fluorescence (TBR > 1,** Figure 3D**). The smallest detectable tissue weights were projected to be 3 mg and 66 mg for high and low dose cohorts, respectively (n = 58 and n = 71, **Figure 3D**), and inversely correlated with panitumumab-IRDye800 dose per kg body weight (R^2^ = 0.57, *P* = 0.007, **Figure 3E**). No difference in NIR fluorescence was observed in tumor tissue resected 1 - 3 days after panitumumab-IRDye800 infusion, indicated by the overlapping confidence intervals of linear regressions (for dose normalized NIR against tissue weight) among three imaging windows (**Figure S1**).

### Histological and molecular correlation

**Figure 4:**
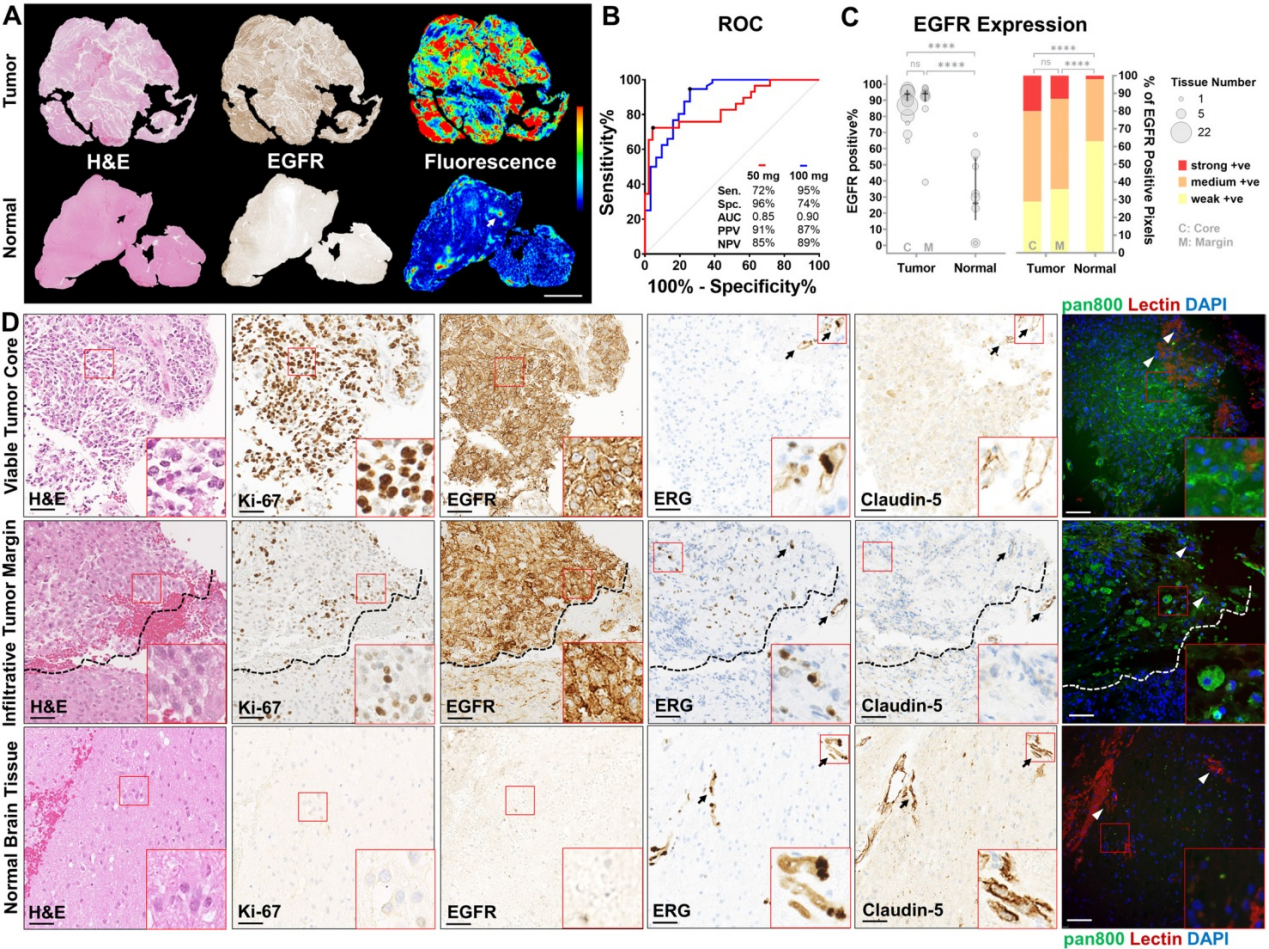
Panitumumab-IRDye800 delivery correlated with tissue histology and molecular biomarkers. (**A**) Presence of viable tumor (H&E staining) and EGFR expression in representative tumor and normal tissue sections and corresponding fluorescence distribution. Scale bar = 2 mm. (**B**) ROC curves for distinguishing tumor against normal tissue with mean fluorescence intensity, reporting sensitivity (Sen.), specificity (Spe.), area under the curve (AUC), positive predictive value (PPV) and negative predictive value (NPV) for each dosing cohort. (**C**) Spatial distribution of immunohistochemical staining positivity and average percentages of three positive staining intensities of histologically confirmed tumor versus normal tissue sections. *P* = 0.9478 (C vs M, bubble plot), *P* = 0.2226 (C vs M, bar graph) and **** *P* < 0.0001 by Chi-square contingency test. (**D**) Micrographs of immunohistochemical staining on cellular and molecular biomarkers including H&E (histology), of Ki-67 (proliferation), EGFR, ETS-related gene (ERG, endothelial nucleus) and Claudin-5 (tight-junction protein), and corresponding fluorescence micrographs of panitumumab-IRDye800 (pan800) delivery in representative sections containing viable tumor core, infiltrative tumor margin and normal brain tissue. *Arrows*: blood vessels; *dashed lines*: infiltration edge; DAPI: nuclear staining; lectin: endothelial membrane staining; Scale bars = 50 µm.

Compared to normal brain tissue, viable tumor tissue overexpressed EGFR (**Figure 4A**), corresponding to a higher mean fluorescence intensity (2.0 vs 0.6, *P* < 0.0001, **Figure S2**). In recurrent tumors which received radiotherapy and chemotherapy, little EGFR expression or nonspecific NIR fluorescence was observed in gliotic brain tissue (**Figure S3**). High specificity (96%) and sensitivity (95%) were achieved in the low and high dose cohorts, respectively, by histological co-localization of the NIR fluorescence (**Figure 4B**). Viable tumor tissue could be identified with positive predictive values (PPVs) and negative predictive values (NPVs) above 85% in both doses while 100 mg dose provided superior area under the curve (AUC) compared to 50 mg (0.90 vs 0.85). EGFR expression was significantly higher (*P* < 0.0001) in viable tumor core (88%, n = 30) and infiltrative tumor margin (91%, n = 39) compared to normal brain (32%, n = 23) (**Figure 4C**). The distribution of strong, medium and weakly positive staining pixels in tumor tissue, at both viable core and infiltrative margin, are significantly different from that in normal brain (*P* < 0.0001). NIR fluorescence microscopy revealed specific cellular distribution of panitumumab-IRDye800 correlated with highly proliferative tumor cells (Ki-67) and positive EGFR expression in histologically confirmed (by H&E staining) viable tumor core and infiltrative tumor margin of high-grade gliomas (**Figure 4D**). In contrast, minimal EGFR expression and fluorescence signal were found beyond the infiltration edge (dashed lines) and in normal brain tissue, indicating panitumumab-IRDye800 is capable of delineating a histologic tumor margin and solving the problem of low negative predictive value in humans. Tight junction protein expression (Claudin-5, endothelial membrane) around blood vessels (ERG, endothelial nucleus) was used to assess the integrity of blood-brain barrier in endothelial cells of the capillary wall. This was reduced in viable tumor core and infiltrative tumor tissue compared to normal brain. Moreover, delivery of panitumumab-IRDye800 beyond blood vessel elements, indicated extravasation of this antibody-dye conjugate across the suspected, compromised blood-brain barrier in tumor tissue.

## Discussion

Panitumumab-IRDye800 is a fully humanized EGFR antibody currently undergoing early phase clinical trials for fluorescence-guided surgery of brain cancers (NCT03510208 and NCT04085887 for adult and pediatric HGGs, respectively). In this phase 1 trial, sub-therapeutic doses (0.6 - 1.6 mg/kg) given to HGG patients preoperatively resulted in no adverse events attributed to the study drug in either dose cohort, which is the same as previously reported adverse event rate (0%) for panitumumab-IRDye800 in the 1.5 - 2.0 mg/kg dose range and lower than the reported 4% rate in the 0.5 - 1.5 mg/kg dose range in 81 patients across four cancer types 26. Fluorescence guided surgical imaging using panitumumab-IRDye800 was able to detect both primary and recurrent IDH-wildtype HGGs in real time with higher resolution, tumor contrast and specificity than the MRI-based navigation. Comparing to a previous trial in HGG using cetuximab-IRDye800 24, the tumor detection threshold using panitumumab-IRDye800 improved by 2 - 3 folds (depending on dose) in fresh tissue, whereas in fixed tissue sections the specificity of panitumumab-IRDye800 was higher than that of cetuximab-IRDye800 by 45% (96% vs 66%, low dose) and 6% (74% vs 70%, high dose) while the sensitivity remained equivalent. The improvement can be contributed to a few factors including higher dye-to-protein ratio (2.0 vs 1.8 37), optimized open-field imaging features (e.g. room light compensation) in intraoperative imaging device and greater number of HGG patients (11 vs 2). Although preclinical studies have the advantage of non-cross-reactive background tissue (no human EGFR expression in mouse brain), the positive predictive values of panitumumab-IRDye800 in this clinical trial were equivalent to the orthotopic rodent studies using IRDye800-labeled anti-EGFR affibody 38.

Surgeons rely constantly on visual feedback to make resection decisions during operations. Thus by providing real-time enhanced tumor contrast, fluorescence imaging has been readily adopted in the clinical practice since the first use of fluorescein during neurosurgery in 1948 39. The sensitivity and specificity of fluorescein in identifying tumor tissue were 80.8% and 79.1%, respectively, in a multicentric prospective phase 2 study 40. The current FDA-approved intraoperative optical imaging agent for glioma surgery, 5-ALA, has been clinically successful with positive predictive values close to and over 90% reported for malignant gliomas 41. For discriminating HGG tissue from healthy brain tissue using 5-ALA, a broad range of sensitivity (70 - 95%) and specificity (43 - 100%) were reported, making a direct comparison with our results challenging 41-44. However, the infiltrative nature of HGGs has brought forth the need for more specific tumor targeting strategies, as false negatives using fluorescein occurred due to lack of fluorescence in areas of diffuse, low-density cellular infiltration 45. Similar challenges are encountered in 5-ALA, where the metabolic pathway that generates the fluorescent PpIX from 5-ALA is also present in normal brain cells, only to a less extent. Moreover, 5-ALA consumption and PpIX production may be highly variable and depend on several factors such as cell type, glucose concentration, and pH 46-48. The limited tumor specificity in the metabolic targeting strategy of 5-ALA is accentuated especially beyond the contrast-enhancing tumor components in glioblastoma where diffuse pink fluorescence from PPIX has been detected in both infiltrating tumor cells and edematous brain tissue 49, resulting in limited imaging contrast that contributes to the low negative predictive values of 5-ALA (16.7% and 43.9% for HGG and GBM, respectively) reported in a prospective phase 2 clinical trial, compared to above 85% for both doses in this clinical trial using panitumumab-IRDye800 50. Indeed it can be difficult to compare the specificity of panitumumab-IRDye800 with 5-ALA at infiltrative tumor margins due to different mapping mechanisms from fluorescence intensity to the color overlay (heatmap vs saturation) and the lack of information on how far out the tumor biopsies were performed with study patients at the margin. Direct comparison between the intraoperative imaging of these two probes in the same patient would elucidate their comparative performance more conclusively in the future. In addition, the optimal timing for PpIX fluorescence imaging may vary among individuals as maximal concentrations of the PpIX metabolite occurred within a range of 1.2 - 7.8 h (median: 4 h), due to differential metabolism rates of 5-ALA in various malignant gliomas 51, 52.

Panitumumab-IRDye800 is benchmarked against the FDA-approved 5-ALA as a fluorescence imaging probe for HGGs in** Table 2**. Armed with a molecular-targeting mechanism, panitumumab-IRDye800 has the potential to provide a more robust tumor specificity and negative predictive value lacking in 5-ALA based on our preliminary results, in addition to other desirable characteristics such as low autofluorescence, better stability and drug delivery implications 12. EGFR protein level was enhanced in glioma tissue and moderate to strong EGFR positivity was reported in 75% of malignant glioma patients, which was the 2^nd^ highest across 20 cancer types analyzed by immunohistochemistry in the Human Pathology Atlas 53. EGFR protein was positively expressed in patients in this trial, and 77% of HGG patients (n = 35) we previously tested had EGFR protein expression in their tumor tissue either equivalent or above the detectable level via NIR imaging using panitumumab-IRDye800 19. EGFR-amplified cells are preferentially located at the infiltrating edge rather than distributed uniformly within GBM tumors 54, thus more likely to provide the higher contrast in order to delineate the tumor tissue from normal brain. Despite its higher molecular weight, NIR fluorescence from panitumumab-IRDye800 was observed beyond the contrast-enhancing tumor core in both intraoperative imaging and *ex vivo* fresh tissue in this study. This was surprising given the unlikely penetration of infiltrating margin of HGG by a monoclonal antibody if the BBB is intact. Further examination under fluorescent microscope revealed panitumumab-IRDye800 fluorescence localized in HGG tumor cells, indicating its cellular delivery across the defective blood-brain barrier rather than merely accumulating in vasculature by the enhanced permeability and retention (EPR) effect. The exact mechanism is unclear and being investigated in a preclinical study. However, an early study on microvessel ultrastructure examined human peritumoral brain tissue under microscope and has shown visible evidence for blood-brain barrier defects in peritumoral brain tissue 55 before it is now known that an expanding neoplastic lesion causes local and distal changes that can directly compromise neuronal viability and vascular function 56. Other studies have found BBB permeability in non-enhancing area around T1 enhancing lesion and that even dynamic contrast enhancing MRI may be inadequate for measuring slower rate of BBB leakage 57, 58. In a preclinical model, the specificity and sensitivity of 5-ALA was lower compared to panitumumab-IRDye800 and the tumor-to-background ratio for panitumumab-IRDye800 was four times greater than that of 5-ALA 18. In the meantime, the workflow disruption from using a hand-held device (SPY-PHI) can be mitigated with an FDA-approved commercial surgical microscope compatible with NIR imaging, such as the KINEVO 900 with INFRARED 800 filter used in this study 10. Furthermore, labeling panitumumab with a fluorophore in the second near infrared window (NIR-II, 1000 - 1700 nm) can potentially push its detectability to more than one centimeter beneath the tissue surface 59. Potentially, future intelligent therapy that integrates precision diagnosis and intraoperative fluorescence imaging guided laser ablation may provide high-precision therapeutic effect for the resection of brain tumors 60. Evaluation of the efficacy and cost-effectiveness of panitumumab-IRDye800 against existing fluorescence-guided resection approaches is necessary for its regulatory approval and adoption in standard clinical practice.

As this is a first-in-human phase 1 study in a limited number of patients, only patient-level evidence is available at this point supporting the safety and feasibility of using panitumumab-IRDye800 for HGG imaging, while a body of study-level evidence and experience has been accumulated for 5-ALA. Additional dosing cohorts may further optimize dose selection and help determine maximum tolerated dose of panitumumab-IRDye800 in future studies. Although no antitumor effect has been reported in preclinical studies with panitumumab-IRDye800 administration, microscopic changes and long term effect from EGFR-targeting imaging probes cannot be ruled out. In addition, as recruitment was prospective on suspected HGGs based on MRI imaging, we were not able to include more Grade III HGGs patients and more diverse HGG disease subtypes, which may shine light on the value of panitumumab-IRDye800 for these indications. The measurement accuracy of T1-MRI contrast of tumor at individual intraoperative interrogation sites can be affected by brain shift, and compensation methods for this effect are currently under investigation 61. It remains to be investigated, for instance, in supratotal resection cases of HGGs, whether panitumumab-IRDye800 can detect regions of even lower tumor cell infiltration than seen in resection specimens in the current study, which would put its sensitivity and specificity to test. Contributing reasons for the wide range (22 - 89%) of reported EGFR expression in HGG tissue in literature include differences in antibodies, tissue processing, staining techniques, and patient populations, as well as methodological differences in scoring of the EGFR tissue staining 15-17. The EGFR expression quantification method used in this study is subject to the IHC staining intensity thresholds adopted. We included weakly positive stainings as positive as they were detectable by panitumumab-IRDye800 imaging in a preclinical study 19. Consequently, if more conservative criteria adopted by some studies in the literature were applied 62, then not all patients in this study may be considered overexpressing EGFR and the percentage of positive EGFR expression may be different accordingly, which can be inferred from the distribution breakdown of the three positive staining levels in this study. Additional future cases and studies will hopefully provide a thorough understanding of the full potentials and limitations of this molecular targeted imaging strategy.

## Conclusions

In summary, this first-in-human dose ranging study showed that panitumumab-IRDye800 is safe and feasible for fluorescence-guided surgery in patients with high-grade gliomas undergoing surgical intervention, and that it has the potential to enhance visualization of HGGs in the contrast-enhancing tumor core as well as the infiltrative tumor margin. The safety, dose, and surgical timing associated with the use of panitumumab-IRDye800 in this study can be used to inform future studies. To determine the clinical value of this intraoperative imaging method in a larger cohort of HGG patients, a phase 2 study is planned to acquire additional supportive evidence of fluorescence-guided surgery with panitumumab-IRDye800 in comparison against white light standard imaging and fluorescence guided surgery with 5-ALA in visualizing tumor margins to guide the extent of resection.

## Supplementary Material

Supplementary figures and tables.

## Figures and Tables

**Table 1:** Patient demographics and medical record.

	Age (years)	Surgical Procedure	Tumor Site	Histopathological Diagnosis	WHO Grade	Maximum Tumor Diameter (cm)	Extent of Resection	IDH1 Status	Tumor History	Previous Chemotherapy	Previous Radiotherapy
**50 mg Cohort**											
Patient 1	55	Awake craniotomy	left temporal	Glioblastoma	IV	5.3	STR	Wildtype	Primary	No	No
Patient 2	42	Craniotomy	left frontal	Anaplastic oligodendroglioma	III	5.0	STR	Mutated	Recurrent	TMZ, CCNU and bevacizumab	Yes
Patient 3	67	Craniotomy	left parietal	Glioblastoma	IV	3.5	NTR	Wildtype	Primary	No	No
Patient 4	72	Craniotomy	left temporal	Glioblastoma	IV	3.7	GTR	Wildtype	Primary	No	No
Patient 5	62	Craniotomy	left frontal	Glioblastoma	IV	6.1	STR	Wildtype	Recurrent	TMZ and bevacizumab	Yes
**100 mg Cohort**											
Patient 6	49	Craniotomy	left frontal	Glioblastoma	IV	1.3	GTR	Wildtype	Primary	No	No
Patient 7	60	Awake craniotomy	right frontal	Glioblastoma	IV	4.4	STR	Wildtype	Primary	No	No
Patient 8	67	Craniotomy	left parietal	Glioblastoma	IV	5.8	STR	Wildtype	Primary	No	No
Patient 9	32	Craniotomy	right temporal	Glioblastoma	IV	2.8	STR	Wildtype	Recurrent	TMZ and CP	Yes
Patient 10	70	Awake craniotomy	left frontal	Glioblastoma	IV	2.9	NTR	Wildtype	Primary	No	No
Patient 11	36	Craniotomy	right temporal	Glioblastoma	IV	7.4	STR	Wildtype	Recurrent	TMZ	Yes

STR = subtotal resection, NTR = near total resection, GTR = gross total resection, TMZ = Temozolomide, CCNU = 1-[2-chloroethyl]3-cyclohexyl-1-nitrosurea, CP = cyclophosphamide.

**Table 2:** Panitumumab-IRDye800 benchmarked against 5-ALA as a clinical fluorescence imaging probe for HGGs.

Characteristics	5-ALA	Panitumumab-IRDye800
Molecular weight 21	131 Da	150 kDa
Tumor-targeting mechanism 63, 64	Metabolism	Immunology
Mode of administration and time to imaging 21, 52, 63	Oral, 2 - 4 hours	IV, 1 - 5 days
Dose 9, 52	20 mg/kg	50 mg & 100 mg
Sensitivity 41-44	70 - 95%	72% & 95%
Specificity 41-44	43 - 100%	96% & 74%
Positive predictive value 41, 44	88 - 100%	91% & 87%
Negative predictive value 41, 50	17 - 91%	85% & 89%
Detectable concentration with clinical imaging systems 35, 65	760 pM	13 pM
Penetrates the blood-brain barrier 18, 19, 38, 41, 66	√	√
Good safety profile in human 21, 67	√	√
Good bioavailability 21, 68	√	√
Long shelf life for storage (unopened bottle) 26, 69	√	√
FDA approved intraoperative imaging systems 10, 11, 64	√	√
Real-time intraoperative guidance with minimal workflow disruption 41	√	
Extensive in-human data on various brain lesions 9, 41, 43, 44, 50	√	
FDA approval for standard clinical practice 12	√	
Low background autofluorescence 11, 63		√
Photo, physically and chemically stable (in solution) 26, 69		√
Predicts drug delivery for antibody therapy 35		√
Cost effective ( < $50K / quality-adjusted life year) 44, 70	√	*

* Not reported.

## Abbreviations

5-ALA: 5-aminolevulinic acid
AUC: area under the curve
BOLD: blood oxygenation level dependent
C: core
CCNU: 1-[2-chloroethyl]3-cyclohexyl-1-nitrosurea
CE: contrast-enhancing
CNR: contrast-to-noise ratio
CP: cyclophosphamide
CUSA: cavitron ultrasonic surgical aspirator
DAPI: 4', 6-diamidino-2-phenylindole
DLT: dose limiting toxicity
EGF: epidermal growth factor
EGFR: epidermal growth factor receptor
EPR: enhanced permeability and retention
ERG: ETS-related gene
FDA: food and drug administration
FFPE: formalin fixed and paraffin embedded
GBM: glioblastoma
GTR: gross total resection
H&E: hematoxylin and eosin
HGG: high-grade glioma
IDH: isocitrate dehydrogenase
IHC: immunohistochemistry
IND: investigational new drug
IR: infrared
IRB: institutional Review Board
M: margin
MFI: mean fluorescence intensity
fMRI: functional magnetic resonance imaging
iMRI: intraoperative magnetic resonance imaging
NCT: national clinical trial
NE: non-enhancing
NExT: NCI (National Cancer Institute) experimental therapeutics
NIR: near-infrared
NPV: negative predictive value
NTR: near total resection
OR: operating room
PpIX: protoporphyrin IX
PHI: portable handheld imager
PPV: positive predictive value
ROC: receiver operating characteristics
ROI: region of interest
SAE: serious adverse event
SEM: standard error of the mean
STR: subtotal resection
TBR: target-to-background ratio
TMZ: temozolomide
T1 + C: T1 + contrast
WHO: world health organization
