# Supplementary material for: EGFR-targeted intraoperative fluorescence imaging detects high-grade glioma with panitumumab-IRDye800 in a phase 1 clinical trial
Source: Theranostics. 2021 May 21;11(15):7130–43. doi: 10.7150/thno.60582 (PMC8210618; doi:10.7150/thno.60582)

Supplementary information

Table S1. Severity and attribution of adverse events

	Adverse Event	Grade	Attribution	SAE*	DLT†
**50 mg Cohort**					
Patient 1					
Day 16	Pain	1	Unrelated	No	No
Patient 2					
Day 16	Fatigue	1	Unrelated	No	No
Day 16	Wound infection	1	Unrelated	No	No
Day 16	Localized edema	1	Unrelated	No	No
Day 30	Arthralgia	1	Unrelated	No	No
Patient 3					
Day 30	Edema limbs	2	Unrelated	No	No
Patient 5					
Day 16	Confusion	2	Unrelated	No	No
Day 30	Confusion	1	Unrelated	No	No
**100 mg Cohort**					
Patient 7					
Day 16	Memory impairment	1	Unrelated	No	No
Patient 8					
Day 2	Vestibular disorder	1	Unrelated	No	No
Day 2	Hearing impaired	1	Unrelated	No	No
Day 2	Concentration impairment	1	Unrelated	No	No
Day 2	Gait disturbance	1	Unrelated	No	No
Day 2	Hypertension	1	Unrelated	No	No
Day 16	Vestibular disorder	1	Unrelated	No	No
Day 16	Confusion	1	Unrelated	No	No
Day 16	Memory impairment	1	Unrelated	No	No
Day 16	Muscle weakness right-sided	1	Unrelated	No	No
Day 16	Headache	1	Unrelated	No	No
Day 16	Fatigue	1	Unrelated	No	No
Day 16	Dysphasia	1	Unrelated	No	No
Day 30	Confusion	1	Unrelated	No	No
Day 30	Memory impairment	1	Unrelated	No	No
Day 30	Muscle weakness right-sided	1	Unrelated	No	No
Day 30	Headache	1	Unrelated	No	No
Day 30	Fatigue	1	Unrelated	No	No
Day 30	Dysphasia	1	Unrelated	No	No
Patient 10					
Day 1	Seizure	3	Unrelated	No	No
Day 1	Muscle weakness upper limb	1	Unrelated	No	No
Day 1	Fatigue	1	Unrelated	No	No
Day 1	Myalgia	1	Unrelated	No	No
Day 1	Tinnitus	1	Unrelated	No	No
Day 1	Dysphasia	1	Unrelated	No	No
Day 1	Seizure	1	Unrelated	No	No
Day 2	Seizure	1	Unrelated	No	No
Day 30	Seizure	1	Unrelated	No	No
Day 30	Seizure	1	Unrelated	No	No
Day 30	Seizure	1	Unrelated	No	No
Day 30	Seizure	1	Unrelated	No	No
Day 30	Seizure	1	Unrelated	No	No
Patient 11					
Day 30	Headache	1	Unrelated	No	No

* SAE = serious adverse event; † DLT = dose limiting toxicity

Figure S1. Fresh tissue fluorescence at three different imaging windows. Linear regression (with 95% confidence intervals) of mean fluorescence intensity (MFI) normalized to panitumumab-IRDye800 dose (mg/kg) against tissue weight of fresh tissue pieces removed 1 day, 2 days and 3 days after panitumumab-IRDye800 infusion.

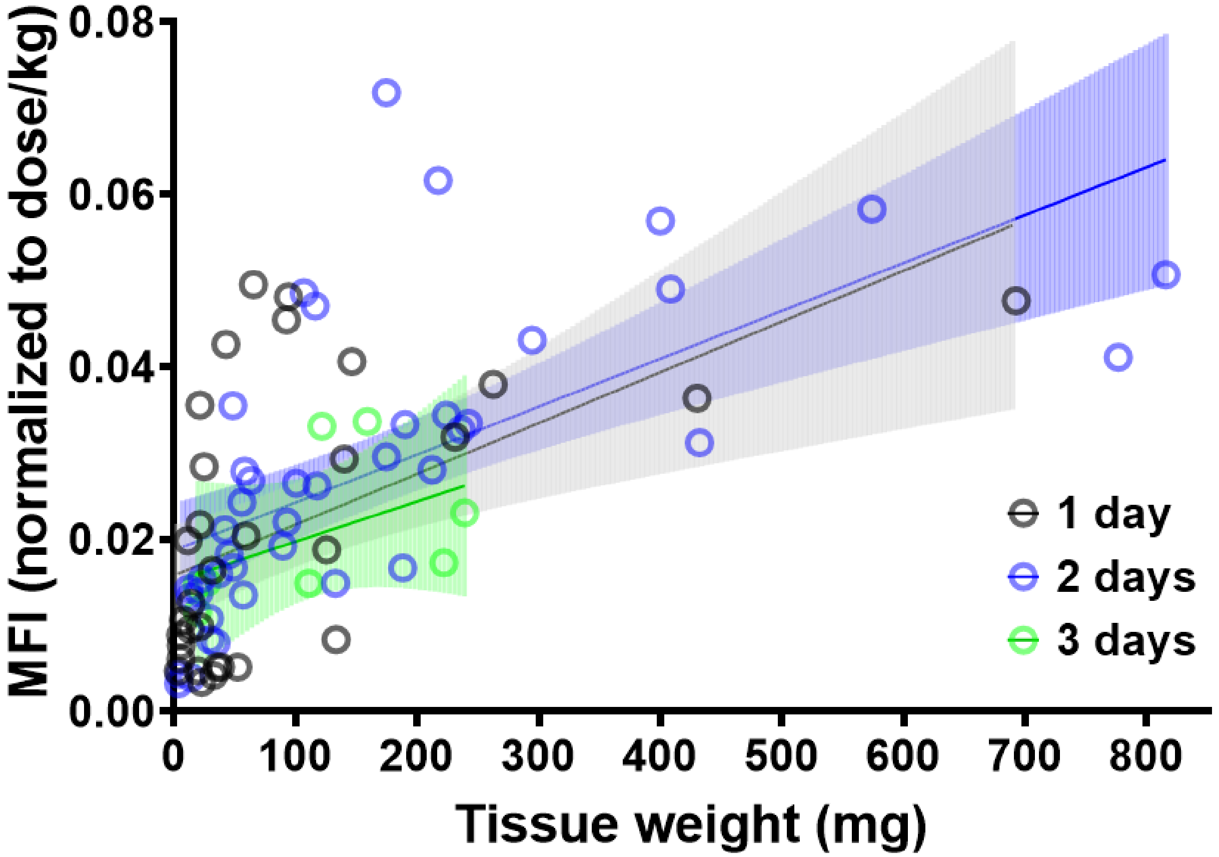

Figure S2. MFI (*left y-axis*) of tumor (T) and normal (N) tissue sections and tumor TBR (*left y-axis*) for low and high dose cohorts. *Dotted lines*: MFI cutoff values (0.5545, red; 0.7445, blue) for maximal specificity and sensitivity of respective ROC curves in **Figure 4B**; $P < 0.0001$ (50 mg T vs 50 mg N; 100 mg T vs 100 mg N; 50 mg T vs 100 mg T) and $P = 0.018$ (50 mg N vs 100 mg N) for MFI comparisons; $P = 0.0035$ (50 mg TBR vs 100 mg TBR). Statistical test: unpaired t-test with Welch's correction.

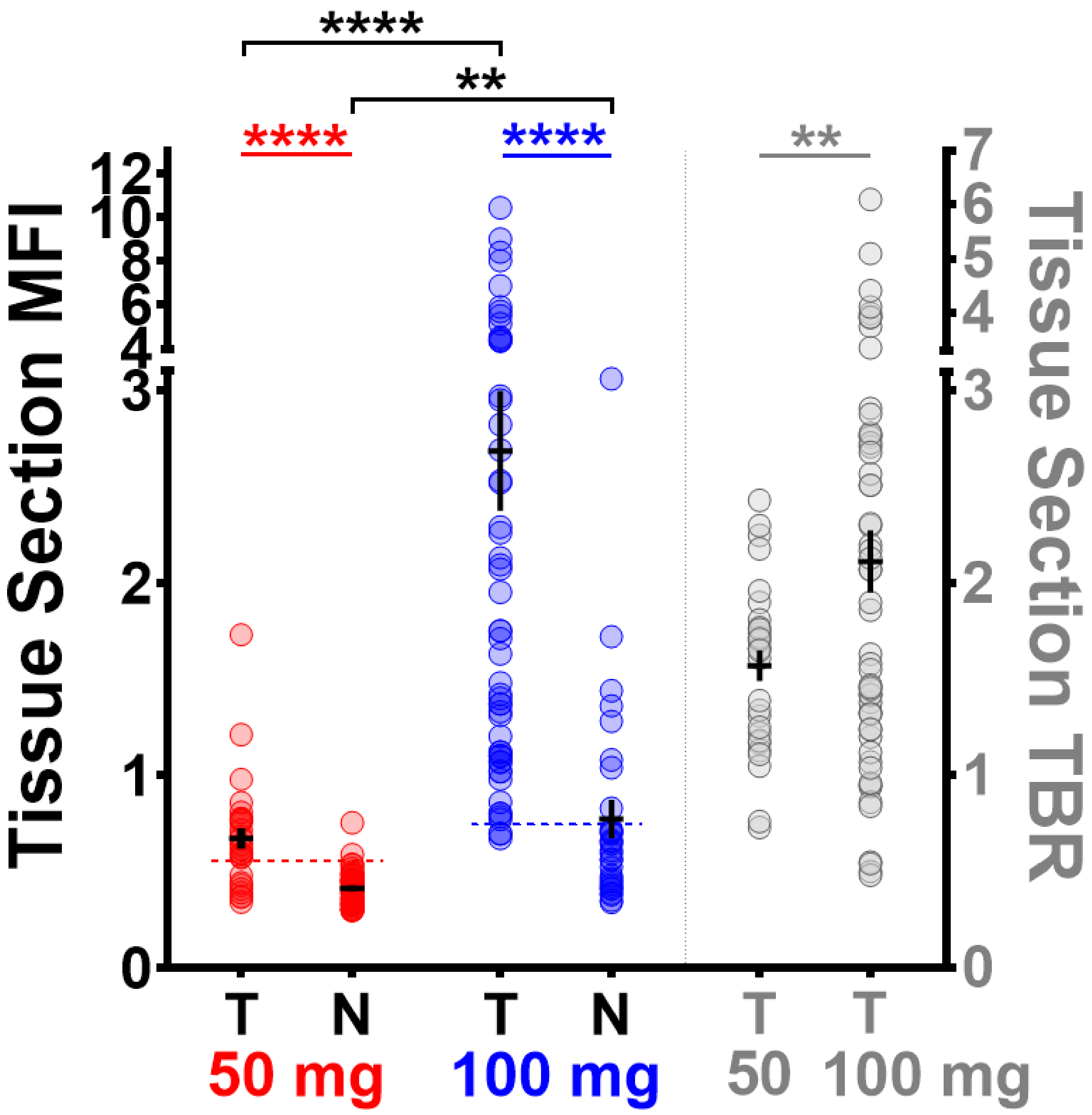

Figure S3. NIR fluorescence in gliotic brain tissue. Histological and immunohistochemical stainings of resected HGG tissue containing gliosis (*dotted outlines*).

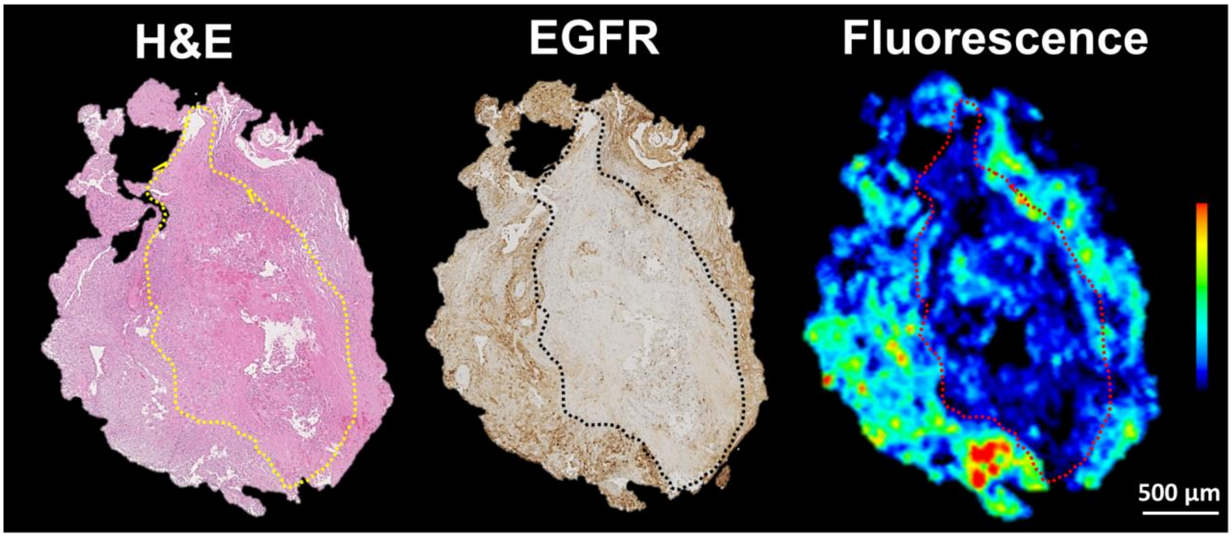

Supplement: Supplementary file 1 — Supplementary figures and tables. [file thnov11p7130s1.pdf]
